# GABA_A_ binding correlates with high-frequency EEG: a possible proxy for depolarization in traumatic brain injury

**DOI:** 10.1093/braincomms/fcag145

**Published:** 2026-04-27

**Authors:** Sudhin A Shah, Ludvik Alkhoury, Isabelle Martin, Ana Radanovic, Giacomo Scanavini, Seyed Hani Hojjati, Gloria C Chiang, Tracy A Butler, Yeona Kang, Keith W Jamison, Amy Kuceyeski, Nicholas D Schiff

**Affiliations:** Department of Radiology, Weill Cornell Medicine, New York, NY 10065, USA; Department of BMRI & Neurology, Weill Cornell Medicine, New York, NY 10065, USA; Department of Radiology, Weill Cornell Medicine, New York, NY 10065, USA; Department of Radiology, Weill Cornell Medicine, New York, NY 10065, USA; Department of Radiology, Weill Cornell Medicine, New York, NY 10065, USA; Department of Radiology, Weill Cornell Medicine, New York, NY 10065, USA; Department of Radiology, Weill Cornell Medicine, New York, NY 10065, USA; Department of Radiology, Weill Cornell Medicine, New York, NY 10065, USA; Department of Radiology, Weill Cornell Medicine, New York, NY 10065, USA; Department of Mathematics, Howard University, Washington, DC 20059, USA; Department of Radiology, Weill Cornell Medicine, New York, NY 10065, USA; Department of Computational Biology, Cornell University, Ithaca, NY 14850, USA; Department of Radiology, Weill Cornell Medicine, New York, NY 10065, USA; Department of Computational Biology, Cornell University, Ithaca, NY 14850, USA; Department of BMRI & Neurology, Weill Cornell Medicine, New York, NY 10065, USA

**Keywords:** neuroimaging, cognitive recovery, biomarkers of impairment, neocortical circuits

## Abstract

Traumatic brain injury (TBI) frequently results in long-term cognitive and functional deficits, yet routine diagnostic tools often fail to capture the diffuse network dysfunction and underlying neurochemical changes that contribute to poor recovery. Resting-state EEG is sensitive to injury-related abnormalities in neural oscillations, while [^11^C]flumazenil PET quantifies gamma-aminobutyric acid (GABA_A_) receptor availability—a key determinant of inhibitory tone and cortical synchronization—but these modalities are rarely integrated. Linking EEG spectral features to molecular measures of GABA_A_ function may provide translational biomarkers that bridge non-invasive neurophysiological findings with underlying neurochemical status. The primary aim of this study was to determine whether resting-state EEG spectral features, particularly in the high-frequency beta and gamma bands, track longitudinal changes in GABA_A_ receptor availability measured with [^11^C]flumazenil PET in individuals recovering from TBI. A secondary aim was to characterize the persistence of low-frequency abnormalities (increased delta, reduced alpha) over the first year of recovery and explore their potential relevance as non-invasive markers of network dysfunction. We analysed EEG data from 68 subjects with TBI and 75 non-brain-injured controls; longitudinal follow-up EEG data were available for 37 TBI participants and 20 non-brain-injured controls. We found that the TBI subjects exhibited significantly higher delta power and lower alpha power; this remained at the chronic visit. Among the longitudinally studied subjects, a subset of seven TBI participants and six non-brain-injured controls were studied with [^11^C]flumazenil PET data. We found strong positive correlations between longitudinal changes in [^11^C]flumazenil PET measured GABA_A_ receptor availability and concurrent changes in EEG high beta (*r*^2^ = 0.79, *P* < 0.01) as well as low and high gamma power (*r*^2^ = 0.71, *P* < 0.05; *r*^2^ = 0.77, *P* < 0.01) in TBI subjects for the ‘eyes-open’ condition. These exploratory findings provide preliminary evidence that GABA_A_ receptor availability, measured via [^11^C] flumazenil PET, is associated with high-frequency EEG power in TBI. This PET–EEG coupling may reflect underlying changes in excitatory–inhibitory network balance consistent with restoration of fronto-striatal arousal and neuronal membrane ‘tone’ under the mesocircuit model, although the small sample size of the cohort with multimodal measurements warrants cautious interpretation and further replication. Nonetheless, the observations in this cohort are consistent with a key role of increasing inhibitory activity across fronto-striatal neurons and networks in recovery from TBI.

## Teaser

Shah *et al.* examined GABA_A_ receptor availability, measured by flumazenil PET, and resting EEG power across traumatic brain injury recovery. Longitudinal increases in receptor availability correlated positively with high-frequency EEG power, supporting EEG spectral markers as indicators of cortical dysfunction, network disintegration and prognostic recovery trajectories after traumatic brain injury.

## Graphical Abstract

**Graphical Abstract:**
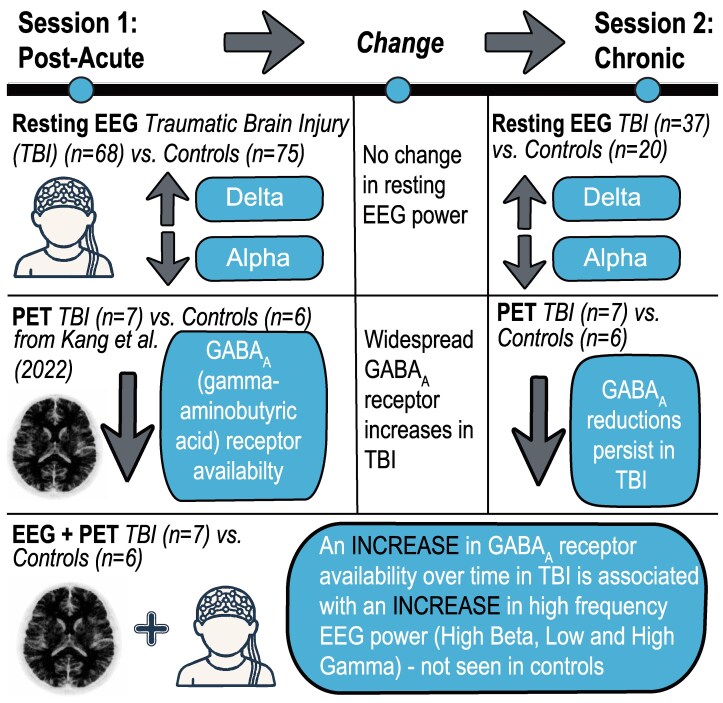

## Introduction

Traumatic brain injury (TBI) remains a leading cause of long-term cognitive and functional disability, yet current diagnostic approaches often fail to capture the diffuse and dynamic nature of its neural consequences.^1^ While conventional neuroimaging can identify structural abnormalities, such as hematomas and oedema, it often fails to capture functional changes within and across neuronal types and networks.^2-4^ These include disruptions in local connectivity, synaptic signalling, receptor down-regulation, network coordination and the intrinsic cellular balance of inhibitory and excitatory input in the setting of deafferentation. Though these neurobiological disturbances are not detected with standard imaging, they have strong associations with clinical outcomes.^5-7^

In TBI, resting EEG consistently reveals a spectral shift towards increased low-frequency power (delta) and reduced alpha power–patterns linked to axonal disconnection, thalamocortical dysrhythmia and impaired cognitive function.^8-11^ While these shifts of low-frequency power are well established, far less is known about the status of high-frequency rhythms, particularly in the beta–gamma range, which are strongly shaped by gamma-aminobutyric acid (GABA_A_)-mediated inhibition^12,13^ and can reflect recovery of inhibitory timing within networks.^14,15^ In healthy populations and other neurological disorders, cross-sectional positron emission tomography (PET) and magnetic resonance spectroscopy (MRS) studies have linked GABAergic markers to oscillatory activity in EEG/MEG, especially in the gamma band.^14,16-18^

Several PET and SPECT radioligands have been developed to interrogate the GABA_A_ receptor system following brain injury. The most extensively validated in humans is [^11^C]flumazenil (FMZ), which binds the central benzodiazepine site with high affinity and provides a reproducible index of GABA_A_ receptor availability. FMZ PET has been applied in multiple clinical TBI studies to characterize neuronal loss, inhibitory dysfunction and perilesional changes.^5,7^ In preclinical settings, the fluorinated analogue [^18^F]flumazenil has similarly demonstrated reduced binding in injured cortex after controlled cortical impact,^19^ suggesting translational potential, though human TBI applications are not yet available. Additional emerging PET tracers target related components of the GABAergic system. [^18^F]-GE-194, for example, has shown sensitivity to GABA_A_ receptor–associated alterations in *ex vivo* rodent preparations,^20^ but has not been deployed *in vivo* in TBI. Subunit-selective radioligands such as [^11^C]Ro15-4513 provide α5-specific binding and have been used to probe inhibitory signalling in addiction, psychiatry and epilepsy,^21^ yet have no published applications in TBI to date. Related SPECT tracers include [^123^I]iomazenil (IMZ), which binds the same benzodiazepine site (α1/2/3/5-containing GABA_A_ receptors) and serves as a marker of neuronal integrity. IMZ SPECT has been used in several clinical TBI studies, demonstrating reduced uptake in structurally normal-appearing cortex and associations with cognitive deficits and outcome.^3,4,22^

In previous work, Kang *et al.*^7^ reported longitudinal [^11^C]flumazenil PET (FMZ-PET) findings from the assessment of a subcohort of seven msTBI subjects also studied here. In this prior study patterns of changes in GABA_A_ receptor availability compared to healthy controls were consistent with the mesocircuit model of recovery from TBI^23,24^: TBI subjects showed widespread subacute reductions of FMZ BPND across frontal lobes, striatum and thalamus and increased BPND in the globus pallidus; changes in BPND across these regions measured during subacute recovery at the chronic timepoint showed changes further consistent with the restoration of anterior forebrain mesocircuit activity as inferred from receptor availability as well as a persistently lower FMZ BPND remaining in the frontal cortices of TBI subjects compared to non-brain-injured controls. Longitudinal improvements in executive attention in the TBI group were associated with increased receptor availability in bilateral fronto-parietal cortices and the anterolateral thalamus. These findings support a role for GABAergic signalling in cognitive recovery and underscore the potential of [^11^C]flumazenil PET to track large-scale network restoration and therapeutic response following brain injury.^7^ The findings are also consistent with independent FMZ-PET studies that demonstrate reduced GABA_A_ receptor availability after TBI, also most prominently in frontal cortices and thalamus.^2,5^ Selective thalamic neuronal loss on FMZ-PET has also been specifically related to worse functional and executive outcomes.^6^

Although Kang *et al.*^7^ provided evidence for the mesocircuit model of recovery from TBI, which emphasizes the functional re-afferentation of frontal and striatal neurons as a result of increasing corticothalamic activity and circuit mechanisms involving corticostriatopallidal-thalamocortical loops over time, the use of FMZ-PET alone does not allow further inference of changes at the neuronal and network level predicted by the mesocircuit theory.^23,24^ Combined measurement with EEG offers the potential to link the patterns of GABA_A_ receptor availability during recovery to known roles for inhibitory tone in arousal related brain function: (i) increases in intracellular inhibitory tone increases that occur with more aroused brain states reflected in inhibitory post-synaptic potential rates (which exceed excitatory tone in awake states)^25-27^; in healthy brains, neocortical intracellular function during wakefulness is characterized by a ‘high conductance’ state of intense synaptic bombardment,^25^ producing a depolarized membrane and increased ‘tone’. And, (ii) the role of GABAergic tone at the network level in providing inhibitory timing that shapes network function (and is directly reflected in beta and gamma rhythm enhancement, reviewed in McCormick *et al.*^15^); while such activity is largely balanced between excitation and inhibition at the network level,^28^ inhibitory post-synaptic potentials control the timing and organization of high-frequency network activity above 10 Hz^29^ and reflect dominance of activated EEG states by inhibitory conductances. Network inhibitory timing is reflected in the higher frequency beta and gamma band power of the EEG.

Here, we compare resting-state EEG and [^11^C]flumazenil PET measurements to test the association of high-frequency beta and gamma bands with the parallel recovery of GABA_A_ receptor availability in the months following TBI demonstrated by Kang *et al.*^7^ We test the mechanistic hypothesis that high-frequency oscillations may be a ‘proxy’ for increased resting wakeful inhibitory membrane tone via the indirect assessment of GABA_A_ binding across whole brain structures. We further assess whether characteristic low-frequency abnormalities in TBI (increased delta, reduced alpha) persist over the first year of recovery. By linking functional and biochemical measures, this work aims to identify EEG signatures that could serve as non-invasive proxies for neurochemical status, with direct implications for the interpretation of clinical EEGs and the monitoring of recovery trajectories after TBI.

Accordingly, the primary aim of this study is to determine whether resting-state EEG spectral features, particularly in the high-frequency beta and gamma bands, track longitudinal changes in GABA_A_ receptor availability measured with [^11^C]flumazenil PET in individuals recovering from TBI. The secondary aim is to characterize the persistence of low-frequency abnormalities (increased delta, reduced alpha) over the first year of recovery and explore their potential relevance as non-invasive markers of network dysfunction. Resting-state EEG is commonly collected in both eyes-open and eyes-closed conditions, as each engages partially distinct neural networks. Eyes-closed rest increases posterior alpha power and reflects an internally oriented, low-arousal state, whereas eyes-open rest suppresses alpha and increases beta/gamma activity linked to sustained attention, visual processing and fronto-parietal network engagement.^30^ Given that TBI can disrupt attentional control and sensory integration, collecting both conditions allows for a more comprehensive assessment of neural function and for cross-state consistency checks to mitigate the influence of transient arousal fluctuations.

The primary outcome was the strength and direction of the longitudinal association between changes in high-frequency resting-state EEG power (high beta and gamma bands) and changes in GABA_A_ receptor availability measured with [^11^C]flumazenil PET. We hypothesized that increases in GABA_A_ receptor availability over time would be positively correlated with increases in high-frequency EEG power during the eyes-open resting condition.

## Materials and methods

### Participants

Participants were 68 adults with a history of TBI and 77 non-brain-injured controls (see Table 1). Participants in this current study were drawn from a larger longitudinal study using neuropsychological clinical measures, EEG, eye-tracking, PET and MRI; selected studies have previously been published.^31-33^ Control subjects had no prior brain injury or neurological disorder and no substance abuse within one year prior to study enrolment. Recruitment of non-brain-injured controls was conducted through publicly posted flyers, online platforms such as Craigslist, and informal networks of the research team. TBI subjects were recruited via physician referral from Emergency Departments in the New York City metropolitan area. In participants with TBI, the first session occurred during the post-acute period (S1, post-acute period: mean of 4.5 months; 1.4 SD) and the second visit occurred during the chronic stage of recovery (S2, chronic period: mean of 12.5 months; 2.0 SD). No significant difference is noted in the time between visits for controls and TBI (Welch’s *t*-test: *t*(25.7) = 0.62, *P* = 0.54), or the ages of the participants in each group (Welch’s *t*-test: *t*(133.9) = −1.48, *P* = 0.14). TBI subjects ranged from complicated mild to moderate-severe levels of injury. Subjects with complicated mild injury had intracranial neuroimaging abnormalities on acute CT/MRI scan. Those with moderate-severe brain injury had at least one of the following: post-traumatic amnesia for more than 24 h, loss of consciousness for at least 30 min, and Glasgow Coma Scale (GCS)^34^ in the Emergency Department of <13. All participants spoke English and were able to provide informed consent and complete questionnaires and cognitive testing. We excluded subjects who had a history of alcohol or substance use disorder, and visual, auditory, pre-existing neurological disorders and/or motor impairments that would interfere with cognitive testing. All procedures were approved by the institutional review board (IRB) of Weill Cornell Medicine.

**Table 1:** Demographics

	Full cohort EEG Session 1		Full cohort EEG Session 2		Subcohort EEG + PET Session 1		Subcohort EEG + PET Session 2	
	TBI	Controls	TBI	Controls	TBI	Controls	TBI	Controls
Sample; *n*	68	75	37	20	7	6	7	6
Age (years); mean ± SD	48.4 ± 19.6	43.5 ± 17.0	48.0 ± 19.1	46.6 ± 14.6	48.6 ± 9.2	48.5 ± 10.2	48.6 ± 9.2	48.5 ± 10.2
Female; *n*/%	21/32%	30/40%	11/30%	6/30%	2/29%	2/33%	2/29%	2/33%
Male; *n*/%	47/68%	45/60%	26/70%	14/70%	5/71%	4/67%	5/71%	4/67%
Education (years); mean ± SD	14.9 ± 3.0	18.0 ± 2.54	14.6 ± 3.1	18.1 ± 2.7	15.9 ± 3.4	17.7 ± 1.5	15.9 ± 3.4	17.7 ± 1.5
Injury severity (GCS); *n*/%								
Complicated mild (13–15)	48/71%	–	25/68%	–	4/57%	–	4/57%	–
Moderate (9–12)	7/10%	–	4/11%	–	2/27%	–	2/27%	–
Severe (3–8)	13/19%	–	8/22%	–	1/14%	–	1/14%	–
Injury type; *n* (RTA/fall/assault/other)	29/28/8/3^a^	–	17/12/6/2^a^	–	4/2/1/0	–	4/2/1/0	–
GOSE categories; % (severe disability [3–4]/moderate disability [5–6]/good recovery [7–8])	18.9/27.0/54.1	–	18.8/28.1/53.1	–	14.3/42.9/42.9	–	14.3/0/85.7	–
Change in GOSE; % (improved/declined/no change/no data)	–	–	29.7/27.0/29.7/13.5	–	–	–	71.4/0/28.6/0	–
Time since injury (months); mean ± SD	4.5 ± 1.4	–	12.5 ± 2.0	–	4.0 ± 1.0	–	14.4 ± 3.1	–
Time between sessions (months); mean ± SD	–	–	7.8 ± 2.0	7.3 ± 3.5	–	–	10.4 ± 3.5	5.5 ± 0.8

Subject demographics and injury characteristics.

GCS, Glasgow Coma Scale; GOSE, Glasgow Outcome Scale—Extended; RTA, Road traffic accident; SD, standard deviation.

^a^ *n* = 2 were penetrating TBIs.

### EEG data acquisition and analysis

EEG data were recorded using a 128-channel HydroCel Geodesic Sensor Net (EGI, Eugene, OR)^35^ equipped with saline-based sensors. Electrode impedances were maintained below 75 kΩ at the start of each recording session, consistent with the manufacturer’s guidelines. Signals were acquired at either 250 Hz or 1000 Hz sampling rates. The RANSAC algorithm^36^ was applied to detect bad channels that were then reconstructed via linear interpolation (we used the implementation provided in Appelhoff *et al.*^37^). The data were then re-referenced to the average of the right and left mastoids. Since some datasets were originally sampled at 1000 Hz, the data were resampled to 250 Hz when necessary to ensure consistency across recordings. To eliminate the power line noise, a notch filter at 60 Hz was applied, followed by band-pass filtering between 0.5 and 80 Hz using a 4th-order Butterworth filter.

Subsequently, 90-s eyes-open and eyes-closed epochs were extracted for each participant, except for one participant, for whom only 60 s was available.

Power spectral density estimates were computed using the Welch method^38^ (PSD resolution of 0.33 Hz), averaging over 3-s epochs. The absolute power was calculated (by adding up the power of all PSD bins) for the following frequency bands: delta (1–4 Hz), theta (4–8 Hz), alpha (8–12 Hz), low beta (12–22 Hz), high beta (22–30 Hz), low gamma (30–50 Hz) and high gamma (50–55 and 65–80 Hz), with the 55–65 Hz band excluded to avoid residual line noise artefacts. Relative power was then calculated by dividing the power in each band by the total power in the 0.5–80 Hz range, excluding the 55–65 Hz segment.

### Flumazenil PET

We measured brain GABA_A_ receptor binding using PET imaging with the radioligand ethyl 8-fluoro-5,6-dihydro-5-[^11^C]methyl-6-oxo-4H-imidazo[1,5-a][1,4]benzodiazepine-3-carboxylate, or [^11^C]-flumazenil (FMZ). Binding potential (BPND) to GABA_A_ receptors was extracted from the FMZ-PET images using PMOD software^39^ that implements the Logan graphical model^40^ with pons acting as reference region. See Kang *et al.*^7^ for details on the PET acquisition and processing. In Kang *et al*.^7^ a simplified reference tissue model was used to estimate binding potential, whereas here, Logan graphical analysis was used–internal comparisons of the models are highly consistent with each other (unpublished data). Head motion was quantified using framewise displacement (mm), calculated from realignment parameters after excluding the first four noisy frames.

### Statistical analysis

**Figure 1:**
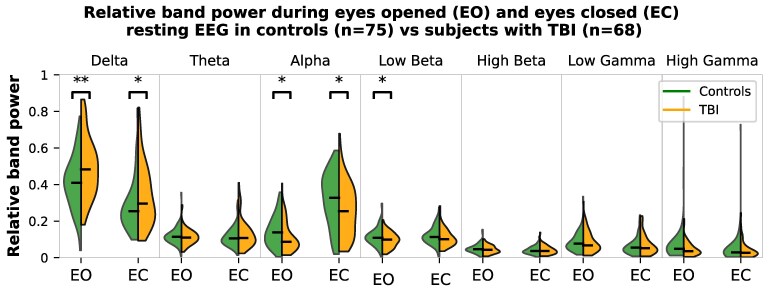
**Relative band power during resting EEG.** Relative band power (absolute power in band divided by total power across all bands) in delta, alpha, and low beta is different between controls and subjects with TBI; Welch’s *t*-test results are indicated with asterisks—uncorrected *P*-value <0.05* and *P*-value <0.01**. Following correction for multiple comparisons using the Benjamini–Hochberg method, only delta band EO remains significant—corrected *P*-value < 0.05.

**Figure 2:**
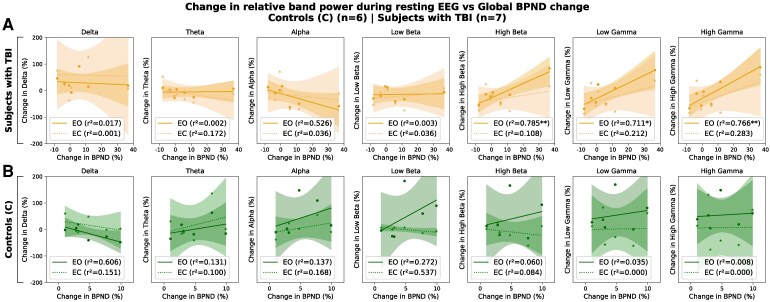
**Relationship between change in resting EEG relative band power and change in FMZ-PET binding potential (BPND).** Change in resting-state EEG band power (%ΔEEG) from Session 1 to Session 2 is plotted against the corresponding % change in regional BPND (%ΔBPND; global, frontal, thalamic), stratified by group (TBI (**A**), control (**B**)) and eye state (EO, EC) across seven canonical frequency bands. For each panel, we fit an ordinary least squares model, %ΔEEG = *β*_0_ + *β*_1_·%ΔBPND + *ε*, and test *H*_0_: *β*_1_ = 0 (two-sided). Points are individuals; lines show the fitted regression with 95% confidence bands. Legends report *r*^2^, with stars indicating uncorrected slope *P*-values (*P < 0.05, **P < 0.01, *P < 0.001). Multiple comparisons were controlled using Benjamini–Hochberg FDR applied once across all 84 tests (7 bands × 2 groups × 2 eye conditions × 3 regions); under this procedure, no tests were significant.

Two-sample Welch *t*-tests^41^ are used to compare the means of variables across control and TBI cohorts, accounting for differences in sample sizes and being conservative regarding the variances of the two groups. Two-sample paired *t*-tests are used when comparing two different sessions (S1 and S2) from within the same cohort; see Supplementary Fig. 1. The Benjamini–Hochberg procedure^42^ is applied to control the false discovery rate (FDR) of multiple comparisons–see Figs 1 and 2.

We assessed group differences in resting-state relative EEG band power with a three-way repeated-measures ANCOVA. Frequency band (delta, theta, alpha, low beta, high beta, low gamma, high gamma) and eyes-open versus eyes-closed conditions were specified as within-subject factors, group (controls versus TBI) as a between-subjects factor and age as a continuous covariate. Subjects were included as blocking factors to account for intra-subject correlation, and sums of squares were computed using a Type II criterion to yield marginal tests of main effects that are invariant to model entry order.^43^

We used the ordinary least squares (OLS) regression to model the relationship between the per cent change in BPND and the per cent change in resting EEG. The significance test on the estimated regressor (Fig. 2) is performed using a one-sample *t*-test with the null hypothesis set to zero. To capture monotonic associations, we also computed Spearman's rank correlation (*ρ*)^44^ and its two-tailed *P*-values.

### Study design and outcomes

#### Primary outcome

Strength and direction of the longitudinal association between per cent change in high-frequency EEG power (high beta and gamma bands) and per cent change in [^11^C]flumazenil PET GABA_A_ receptor binding potential (BPND) between post-acute and chronic recovery timepoints in participants with TBI.

#### Secondary outcomes

(i) Group-level differences in low-frequency EEG power (delta, alpha) between TBI and controls at each timepoint; (ii) stability of EEG power across sessions.

## Results

### Relative EEG band power

A three-way repeated-measures ANCOVA on relative EEG band power—controlling for age and including fixed subject intercepts to account for the non-independence of repeated measures—revealed a highly significant main effect of frequency band (*F*(61 833) = 488.15, *P* < 0.001) but no overall EO versus EC effect (*F*(11 833) = 0.00, *P* = 1.00) or global group difference between controls and TBI patients (*F*(11 833) = 0.03, *P* = 0.87). There was a robust Band × Condition interaction (*F*(61 833) = 64.26, *P* < 0.001), indicating that the EO/EC power change varied by frequency band, and a significant Band × Group interaction (*F*(61 833) = 10.11, *P* < 0.001), showing that group differences in power were band-specific. Neither the Condition × Group interaction (*F*(11 833) = 0.00, *P* = 1.00) nor the three-way Band × Condition × Group interaction (*F*(61 833) = 0.70, *P* = 0.65) reached significance, and age did not covary with power (*F*(11 833) = 0.02, *P* = 0.88). These results demonstrate that while overall spectral profiles differ by band and the EO/EC effect is band-dependent, there are no uniform conditions or group main effects, nor does age influence power once those factors are accounted for. We ran Mauchly's test^45^ and confirmed that the assumption of sphericity was met (*W* = 6.07 × 1e6, *P* = 1.00), indicating that Greenhouse–Geisser correction was not required.

### Group differences in EEG band power

Welch’s *t*-tests^41^ (see Fig. 1) revealed that TBI participants had higher delta power than controls during both EO (*t*(135) = −3.13, *P* = 0.002, Cohen’s *d* = 0.54) and EC conditions (*t*(133.9) = −2.00, *P* = 0.048, *d* = 0.35), lower alpha power during EO (*t*(140) = 2.35, *P* = 0.020, *d* = 0.40) and EC (*t*(140.2) = 2.24, *P* = 0.027, *d* = 0.38) and higher low beta power during EO (*t*(138) = 2.39, *P* = 0.018, *d* = 0.41). Following correction for multiple comparisons using the Benjamini–Hochberg method,^42^ only the EO delta difference remained significant (corrected *P* < 0.05). Notably, EO delta had the largest absolute effect size across all contrasts, which likely contributed to its survival after correction.

### EEG stability across sessions

No statistically significant differences between sessions were observed in the 20 controls and 37 TBI who had resting EEG data collected in two sessions (see Supplementary Fig. 1).

### Relationship between EEG and [^11^C]flumazenil PET: changes in GABA_A_ receptor availability and resting EEG band power

We examined the relationship between resting EEG and the binding potential (BPND) of [^11^C] flumazenil PET (see Fig. 2). Since we have previously reported on changes in GABA_A_ receptor availability over the course of recovery following TBI (Kang *et al.*^7^), we specifically focused on relating longitudinal changes in BPND with longitudinal changes in resting EEG measures. This analysis was conducted in a sample of *n* = 7 subjects with TBI and *n* = 6 non-brain-injured controls in whom EEG and BPND data were available at two timepoints. In subjects with TBI these two timepoints spanned the post-acute to chronic recovery periods. Change was defined as the per cent change from baseline to follow-up, calculated as (S2 − S1)/S1 × 100%. We used OLS regression to model the relationship between the per cent change in BPND and the per cent change in resting EEG band power for each frequency band and condition (EO, EC). We note positive relationships between change in global BPND and change in high beta (*r*^2^ = 0.79, *P* < 0.01), as well as low and high gamma (*r*^2^ = 0.71, *P* < 0.05; *r*^2^ = 0.77, *P* < 0.01), but only in subjects with TBI for the EO condition. Supplementary Fig. 2 shows corresponding associations between frontal and thalamic BPND and resting EEG power, with similar effect sizes: frontal and thalamic BPND versus high beta (*r*^2^ = 0.81, *P* < 0.01; *r*^2^ = 0.61, *P* < 0.05), versus low gamma (*r*^2^ = 0.68, *P* < 0.05; *r*^2^ = 0.63, *P* < 0.05) and high gamma (*r*^2^ = 0.72, *P* < 0.05; *r*^2^ = 0.74, *P* < 0.01). To capture monotonic associations, we also computed Spearman's rank correlation (*ρ*) and its two-tailed *P*-values. We note *ρ* of 0.54 (high beta), 0.68 (low gamma) and 0.82 (high gamma) in subjects with TBI; only high gamma had a *P*-value < 0.05. All reported *P*-values are uncorrected for multiple comparisons, and none of the observed correlations survived correction (Benjamini–Hochberg^42^). These results should therefore be interpreted as exploratory and hypothesis-generating. Head motion, quantified as mean framewise displacement after excluding the first four frames, was low and did not differ between sessions (Session 1: 0.84 ± 0.29 mm; Session 2: 0.96 ± 0.41 mm; *P* = 0.66) in subjects with TBI. In addition, in our prior report, we reported that reference region time activity curves showed no differences across sessions, confirming stability of the denominator in binding potential estimates. Notably, significant PET–EEG correlations were observed only in the eyes-open condition, a state that engages visual and attentional networks; this state-specific effect is explored further in the Discussion in the context of potential neural and neurochemical mechanisms.

## Discussion

Here, we examined group differences in resting-state EEG band power, the longitudinal stability of EEG measures from the post-acute to chronic recovery stages, and the relationship between EEG dynamics and GABA_A_ receptor availability measured with [11C]-flumazenil PET. Across both eyes-open and eyes-closed conditions, our full group of TBI participants (*n* = 37) exhibited increased delta power and decreased alpha power compared to controls, with the eyes-open delta effect surviving multiple-comparison correction. Resting-state EEG measures remained stable over time at the group level, suggesting that the marked changes in GABA_A_ receptor binding observed in TBI were not accompanied by large-scale shifts in spectral power detectable at the group level. Against this background of stability, a state-dependence of FMZ BPND and narrowband EEG coupling is measured in the eyes-open condition: recovery of GABA_A_ receptor availability was associated with increases in high-frequency (beta and gamma) EEG power in TBI. Critically, this positive relationship at the individual subject level of increased gamma/beta EEG power and FMZ BPND—despite the overall lack of group change in EEG power over time—provides a precise and predicted physiological correlation in the context of the study: increases in arousal are lawfully linked to increased intraneuronal inhibitory tone (excess inhibitory post-synaptic potentials, IPSPs) within neocortical neurons^25,26,46^ and improved network inhibitory timing.^12,15,47^

Thus, we find preliminary evidence that combined measurement of FMZ BPND and quantitative EEG measures can further mechanistic investigation and inference in the study of recovery from TBI. We extend the prior findings from Kang *et al.*^7^ in the same subcohort of subjects tested here with FMZ PET, which found that re-engagement of FMZ BPND across the anterior forebrain mesocircuit (AFM) is a molecular readout of recovery. These results were consistent with recent observations that direct electrical stimulation of the central lateral thalamus can restore executive attentional function linked to fronto-striatal networks under the same hypothesis.^23,48^ Improvement in fronto-striatal neuronal function is interpreted in the mesocircuit model^7,23,48^ to reflect an overall increase in both excitatory and inhibitory post-synaptic potentials (favouring the inhibitory contribution) associated with the awake state that results in a more depolarized membrane potential with more ‘tone’—a capacity to rapidly and flexibly respond to internal or external sensory stimuli (reviewed in McCormick *et al.*^15^).

Computational and theoretical work shows that gamma-band oscillations originate in interneuron networks, or pyramidal–interneuron loops, that are synchronized by GABA_A_-mediated IPSPs, with oscillation frequency determined primarily by IPSP decay kinetics and the effective membrane time constant.^12^ The restoration of FMZ BPND over time is thus predicted to coincide with band-limited changes in the high-frequency content of the EEG. The observed longitudinal increases in BDZ-sensitive GABA_A_ receptor availability, particularly within frontal cortex, striatum and thalamus, likely thus reflect recovery of inhibitory timing that permits stronger eyes-open β/γ synchronization; this mechanism is further consistent with the previously reported observed gains in executive attention in this subcohort of subjects.^7^ Moreover, increased GABAergic receptor activity measured by FMZ PET has been specifically linked in healthy controls to gamma and beta power in detailed MEG studies of visual cortex.^14^ The state-dependent association of our findings supports a greater prominence of this linkage in the eyes-open condition. Collectively, although this exploratory study is limited to 14 measurements (7 subjects, 2 time points), the physiological robustness of the relationship has supervened over a heterogeneity of varying functional outcomes and pattern of structural brain injuries in this small group (see also Kang *et al.*^7^).These findings suggest further study in larger cohorts as well as several specific translational and basic neuroscience measurements as noted below.

Together, these findings provide converging evidence for a proposed mechanistic link between molecular GABA_A_ receptor changes and electrophysiological correlates of increasing membrane depolarization tone during recovery from TBI.^7,23^ These changes in EEG high-frequency power likely reflect significant increases in synaptic activity at the neuronal level but are not resolved as group-level changes in EEG power over time due to a variety of factors: heterogeneous recovery across the group of TBI subjects, heterogeneity in injury severity and lesion locations, dominance of the signal power by lower frequency bands of delta and alpha activity, individual variation of actual nominal beta and gamma frequency content, which is influenced by GABA receptor subunit types (as seen in studies of monozygotic twins).^49^ All of these factors can be expected to lead to variability in both the direction and magnitude of EEG change. Thus, although no significant group-level changes in EEG band power were observed across sessions, this does not preclude biologically meaningful within-subject dynamics. Consistent with this expectation, only a subset of participants showed improvement on the Glasgow Outcome Scale—Extended (GOSE) (∼30%, with most others remaining stable or declining); given this limited behavioural recovery, the absence of clear group-level change in resting EEG power is not unexpected. PET–EEG coupling analyses do capture individualized trajectories, revealing that participants who showed increased GABA_A_ receptor availability also tended to show increased high-frequency oscillatory activity during eyes-open rest. Such state-specific, individual-level associations can be masked in group averages but may nonetheless carry prognostic or mechanistic significance, particularly in heterogeneous clinical populations. Similar patterns have been reported in other multimodal TBI studies, where EEG–imaging or EEG–cognitive associations were strong at the individual level despite minimal group-level change.^50,51^

Our PET findings extend previous work linking GABA_A_ receptor dysfunction to impairments following TBI. Prior studies have used GABA_A_-targeted radioligands to relate neuronal loss and receptor dysfunction following TBI. In early foundational work, Shiga *et al*.,^2^ Kawai *et al*.^5^ and Abiko *et al*.^3^ demonstrated reduced flumazenil binding in frontal and temporal cortices, and medial thalamus among TBI patients, correlating them with cognitive deficits. Kang *et al*.^7^ extended these findings longitudinally, showing partial recovery of receptor availability over one year, particularly in the anterior forebrain mesocircuit—a network linked to consciousness and cognition.^23^ In a larger sample, Woodrow *et al*.^6^ also showed persistent thalamic neuronal loss years post-injury, associated with poor functional and cognitive outcomes. Ghosh *et al*.^19^ validated these patterns in a rat TBI model of unihemispheric injury patterns, showing down-regulated GABA_A_ binding and increased inflammation on the injured side.

Here, we associate the measurable increase of GABA_A_ receptor availability over the course of recovery from TBI, with restoration of high-frequency beta/gamma EEG activity. The linkage of these two measurements likely reflects two related mechanisms: one at the intracellular level and the other at the network level. TBI results in both structural and functional deafferentation, reducing resting-state membrane depolarization tone^14,18^ as synaptic background activity is depressed following the acute brain injury. The depolarized state of neocortical membranes during healthy wakeful periods is a high-conductance state dominated by inhibitory conductances, and functional re-afferentation with increasing levels of activity is expected to move closer to this baseline during recovery.^25,27,46^ At the network level, GABAergic control across networks linking frontal cortex, striatum and thalamus shapes the dynamics of high-frequency activity, allowing for temporally precise behaviours and information encoding.^29^ Improved inhibitory timing across these networks leads to synchronization in the beta and gamma bands of EEG activity, resulting in increased power within these frequency ranges.^12,15,47^ The observed correlation between GABA_A_ receptor availability and high-frequency activity may thus provide an indirect window on both intracellular GABAergic tone differences over the course of recovery, from post-acute to chronic timepoints and network dynamics, particularly across fronto-striatal neuronal populations and their loop connections via the thalamus. While this association is consistent with mechanistic hypotheses derived from prior pharmacologic and multimodal imaging work, our small PET–EEG subsample necessitates caution in interpretation, and these findings should be viewed as preliminary until replicated in larger, independent cohorts.

The positive correlation of FMZ BPND and EEG beta/gamma power is consistent with several multimodal studies in healthy participants and other populations. Kujala *et al*.^14^ used task-based magnetoencephalography, MEG, during a visual working memory task alongside [^11^C]Flumazenil PET to show that GABA_A_ receptor density is positively correlated with gamma peak frequency and negatively with gamma amplitude in the primary visual cortex, establishing a direct connection between GABAergic inhibition and gamma oscillations. Similarly, Muthukumaraswamy *et al*.^16^ demonstrated that resting GABA concentration (measured with MRS), is positively correlated with peak gamma frequency (measured with MEG) and negatively with fMRI BOLD response amplitude in visual cortex, indicating that GABAergic inhibition influences both oscillatory and haemodynamic brain activity. Baumgarten *et al.*^18^ further highlighted the role of GABAergic modulation in beta oscillations, showing a significant positive correlation between resting beta peak frequencies and endogenous GABA concentrations in the left sensorimotor cortex, as measured by MRS and MEG.

Additionally, Frankle *et al*.^17^ found that pharmacological enhancement of extracellular GABA (measured with [^11^C]Flumazenil PET) with tiagabine increased binding potential and gamma power in frontal EEG, suggesting that dynamic modulation of GABAergic tone, rather than static receptor density, drives changes in cortical synchronization. GABAergic agents like diazepam, zolpidem, gaboxadol and tiagabine robustly modulate both beta and gamma power, with effects varying based on receptor subtype specificity.^52-55^ Collectively, these converging findings suggest that the positive PET–EEG coupling we observed in TBI reflects a biologically plausible mechanism in which increased GABA_A_ receptor availability during recovery may support enhanced high-frequency oscillatory activity.

In addition to PET–EEG relationships, we observed group-level differences in resting EEG power that persisted across the recovery period. Our findings of increased delta power and reduced alpha power are consistent with prior reports^56-58^; these have generally been linked to deafferentation (increased delta marking axonal disconnection)^59^ or functional recovery (improved alpha power correlating with higher levels of cognitive recovery).^33,60^ The selective survival of the EO delta difference after multiple-comparisons correction likely reflects both statistical and physiological factors. Statistically, EO delta power showed the largest standardized effect size across all contrasts, increasing its resilience to false discovery rate adjustment. Physiologically, EO delta activity may be more sensitive to TBI-related network slowing under conditions requiring sustained alertness, when cortical systems must actively suppress low-frequency activity. By contrast, in the EC condition, delta power is generally higher in all participants, potentially reducing the relative difference between groups. Increased delta activity, particularly in central, parietal and frontal regions, is frequently associated with worse cognitive outcomes, as seen in both acute and chronic TBI cohorts.^56^ Conversely, reductions in alpha power, especially in posterior and fronto-parietal regions, are linked to impairments in executive function, attentional regulation and slower recovery trajectories.^57,58^ Some studies have operationalized these changes using composite metrics such as the delta/alpha ratio (DAR) to stratify recovery potential.^33,60^

### Limitations

Among the limitations of our study, it is important to note that [11C]flumazenil BPND is an indirect measure of GABA_A_ receptor availability and does not directly quantify synaptic inhibition or ‘GABAergic tone’. Binding potential reflects the combined influence of receptor density, affinity states and competition with endogenous GABA, each of which may be altered following injury. Accordingly, our interpretation focuses on receptor availability as a proxy measure that may relate to inhibitory network function, while recognizing that additional physiological and neurochemical processes also contribute to the observed PET–EEG relationships.

The observation that PET–EEG correlations were present only in the EO condition may reflect state-dependent neural and neurochemical dynamics. EO rest places greater demands on visual and attentional systems, potentially increasing the contribution of GABAergic inhibitory processes in beta/gamma-generating cortical circuits. High-frequency oscillations in EO are thought to index local cortical processing and inhibitory–excitatory balance in task-relevant networks; thus, variation in GABA_A_ receptor availability may be more directly expressed in EO beta/gamma power. Conversely, EC rest is dominated by posterior alpha oscillations reflecting idling or default-mode processes, which may dilute the relationship between GABAergic availability and higher-frequency oscillations. This state-specific sensitivity may be particularly relevant for TBI, where attentional control and sensory–motor integration are often affected.

There are several other limitations in this study that should be considered when interpreting the results. The PET–EEG coupling results were derived from a small subset of participants (*n* = 7 TBI, *n* = 6 controls), which limits statistical power and precludes broad generalization. We interpret these findings as hypothesis-generating rather than definitive, noting that replication in larger, independent cohorts will be essential to establish the robustness and clinical utility of the observed associations. Additionally, although we examined the relationship between changes in FMZ BPND and EEG power, we cannot definitively establish causality between altered GABA_A_ receptor availability and changes in oscillatory dynamics, as other neurochemical and functional mechanisms also contribute. Resting EEG can be influenced by fluctuations in arousal. Although segments with possible sleep-like activity were not systematically excluded, the PET–EEG analyses relied on within-subject changes under matched testing conditions. Similarly, while we included a relatively large sample of 75 non-brain-injured controls and 68 individuals with TBI at baseline (S1) for the broader search for correlations of the two measures, the longitudinal comparison of resting EEG measures was also limited by a smaller sample size, with only 20 non-brain-injured controls and 37 TBI participants available at S2. While we did not incorporate structural imaging data (e.g. DTI metrics) in the present analyses, our prior work^7^ has shown that accounting for cortical thickness and subcortical volume does not alter the primary PET findings.

### Conclusions and implications

This study, to our knowledge, is the first to longitudinally examine the relationship between resting EEG spectral features and GABA_A_ receptor availability in TBI, integrating accessible electrophysiological measures with molecular imaging in the same individuals. The combination of EEG and PET offers complementary perspectives–EEG detects functional network alterations with millisecond resolution, while PET quantifies underlying neurochemical changes. Together, these modalities identified a pattern of co-varying changes in functional activity across time in our subcohort that is consistent with the recovery of neuronal and network inhibitory processes during the transition from subacute to more chronic phases of recovery within the first 18 months following TBI, predicted by the mesocircuit model. These findings direct future pre-clinical and translational clinical investigations. Rodent models of TBI would allow the precise evaluation of direct correlation of inhibitory tone (restoration of the high-conductance state) within neocortical neurons, network inhibitory timing and behavioural recovery.^61^ Recent human clinical studies of central lateral thalamic deep brain stimulation in moderate to severe TBI demonstrate late recovery of cognitive function (Schiff *et al.*^48^); paired FMZ-PET and quantitative EEG measurements can be effectively obtained from subjects with the indwelling hardware and allow for direct study of regional activation of GABAergic receptors, injury severity and outcomes.

## Supplementary Material

fcag145_Supplementary_Data

## Data Availability

The data that support the findings of this study are available from the corresponding author, upon reasonable request. The code used for our analysis can be found at this link: https://github.com/S-Shah-Lab/GABAABindingCorrelates.
