# Supplementary material for: GABAA binding correlates with high-frequency EEG: a possible proxy for depolarization in traumatic brain injury
Source: Brain Commun. 2026 Apr 27;8(3):fcag145. doi: 10.1093/braincomms/fcag145 (PMC13148767; doi:10.1093/braincomms/fcag145)
Supplement: fcag145_Supplementary_Data [file fcag145_supplementary_data.pdf]

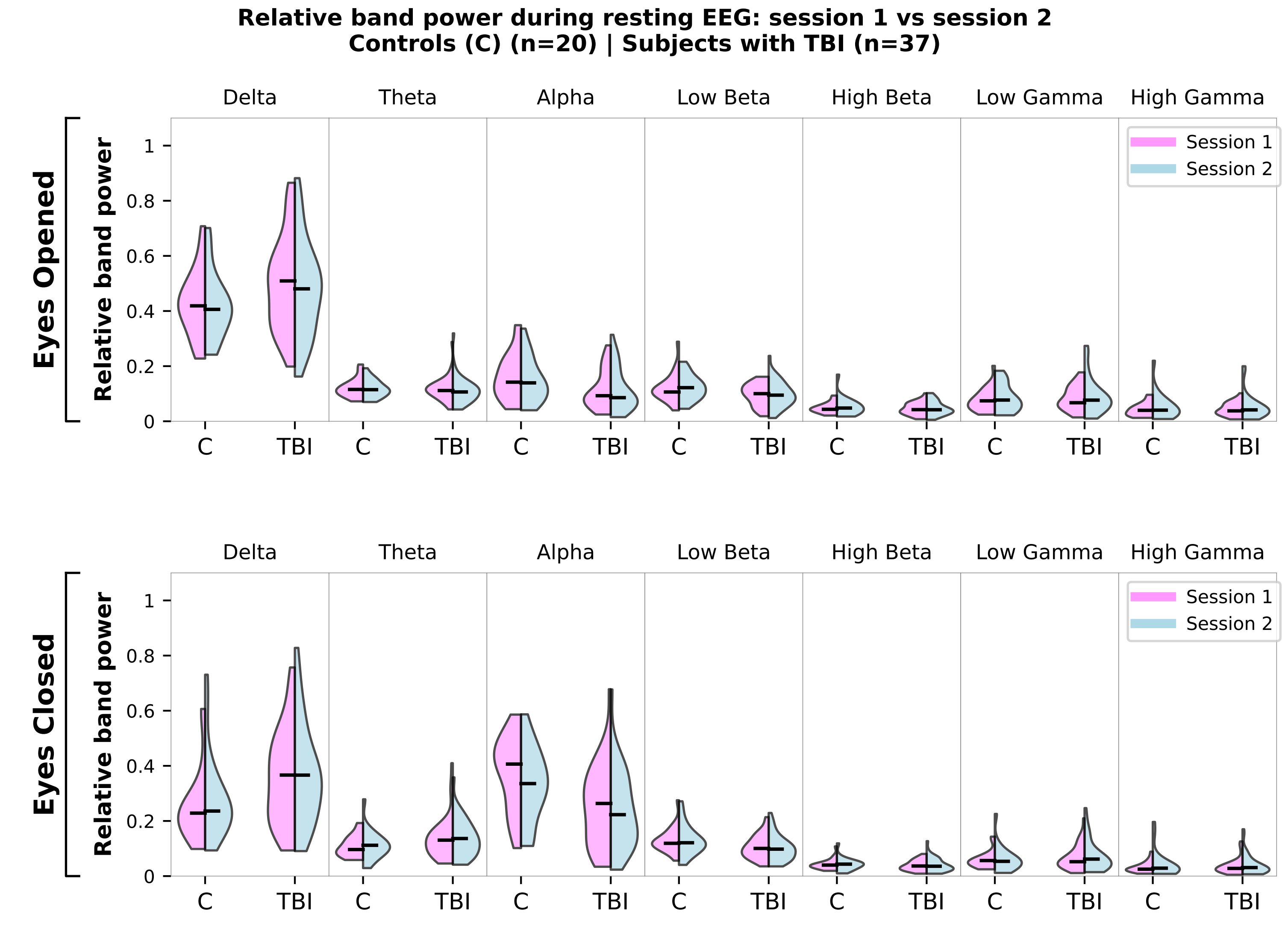
Relative band power during resting EEG: session 1 vs session 2
Controls (C) (n=20) | Subjects with TBI (n=37)
Eyes Opened
Eyes Closed
Relative band power
Delta
Theta
Alpha
Low Beta
High Beta
Low Gamma
High Gamma
Session 1
Session 2
C
TBI
0
0.2
0.4
0.6
0.8
1

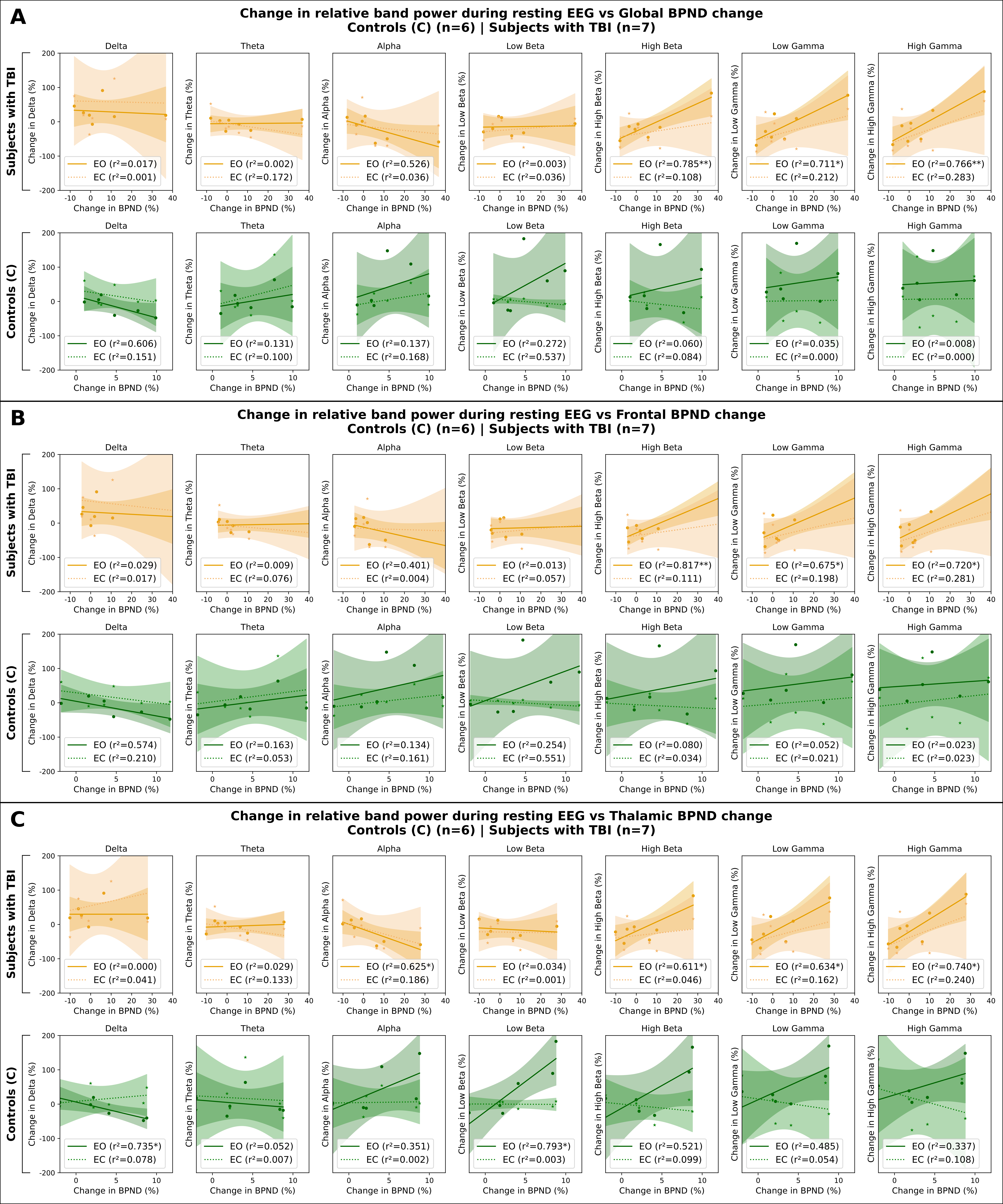

A
Change in relative band power during resting EEG vs Global BPND change
Controls (C) (n=6) | Subjects with TBI (n=7)
Subjects with TBI
Delta
EO (r²=0.017)
EC (r²=0.001)
Theta
EO (r²=0.002)
EC (r²=0.172)
Alpha
EO (r²=0.526)
EC (r²=0.036)
Low Beta
EO (r²=0.003)
EC (r²=0.036)
High Beta
EO (r²=0.785**)
EC (r²=0.108)
Low Gamma
EO (r²=0.711*)
EC (r²=0.212)
High Gamma
EO (r²=0.766**)
EC (r²=0.283)
Controls (C)
Delta
EO (r²=0.606)
EC (r²=0.151)
Theta
EO (r²=0.131)
EC (r²=0.100)
Alpha
EO (r²=0.137)
EC (r²=0.168)
Low Beta
EO (r²=0.272)
EC (r²=0.537)
High Beta
EO (r²=0.060)
EC (r²=0.084)
Low Gamma
EO (r²=0.035)
EC (r²=0.000)
High Gamma
EO (r²=0.008)
EC (r²=0.000)
B
Change in relative band power during resting EEG vs Frontal BPND change
Controls (C) (n=6) | Subjects with TBI (n=7)
Subjects with TBI
Delta
EO (r²=0.029)
EC (r²=0.017)
Theta
EO (r²=0.009)
EC (r²=0.076)
Alpha
EO (r²=0.401)
EC (r²=0.004)
Low Beta
EO (r²=0.013)
EC (r²=0.057)
High Beta
EO (r²=0.817**)
EC (r²=0.111)
Low Gamma
EO (r²=0.675*)
EC (r²=0.198)
High Gamma
EO (r²=0.720*)
EC (r²=0.281)
Controls (C)
Delta
EO (r²=0.574)
EC (r²=0.210)
Theta
EO (r²=0.163)
EC (r²=0.053)
Alpha
EO (r²=0.134)
EC (r²=0.161)
Low Beta
EO (r²=0.254)
EC (r²=0.551)
High Beta
EO (r²=0.080)
EC (r²=0.034)
Low Gamma
EO (r²=0.052)
EC (r²=0.021)
High Gamma
EO (r²=0.023)
EC (r²=0.023)
C
Change in relative band power during resting EEG vs Thalamic BPND change
Controls (C) (n=6) | Subjects with TBI (n=7)
Subjects with TBI
Delta
EO (r²=0.000)
EC (r²=0.041)
Theta
EO (r²=0.029)
EC (r²=0.133)
Alpha
EO (r²=0.625*)
EC (r²=0.186)
Low Beta
EO (r²=0.034)
EC (r²=0.001)
High Beta
EO (r²=0.611*)
EC (r²=0.046)
Low Gamma
EO (r²=0.634*)
EC (r²=0.162)
High Gamma
EO (r²=0.740*)
EC (r²=0.240)
Controls (C)
Delta
EO (r²=0.735*)
EC (r²=0.078)
Theta
EO (r²=0.052)
EC (r²=0.007)
Alpha
EO (r²=0.351)
EC (r²=0.002)
Low Beta
EO (r²=0.793*)
EC (r²=0.003)
High Beta
EO (r²=0.521)
EC (r²=0.099)
Low Gamma
EO (r²=0.485)
EC (r²=0.054)
High Gamma
EO (r²=0.337)
EC (r²=0.108)
Change in BPND (%)
Change in Delta (%)
Change in Theta (%)
Change in Alpha (%)
Change in Low Beta (%)
Change in High Beta (%)
Change in Low Gamma (%)
Change in High Gamma (%)

Figure Titles and Legends:

Supplementary Figure 1: **Longitudinal relative band power during resting EEG.** No changes (paired t-tests) are noted in both controls (n=20) and subjects with TBI (n=37).

Supplementary Figure 2: **Relationship between change in resting EEG relative band power and change in Global (Panel A), Frontal (Panel B), and Thalamic (Panel C) FMZ-PET binding potential (BPND).** EO = eyes opened; EC = eyes closed. From each fit (ordinary-least-squares regression), we report the coefficient of determination (r^2) and two-tailed p value for the slope, overlaying regression lines and 95% confidence intervals on the scatter plots and flagging significance with * p-value <0.05 and ** p-value <0.01. Strong positive relationships are noted for change in high beta and gamma vs change in Global, Frontal, and Thalamic BPND in subjects with TBI for the EO condition (n=7). This is not noted in controls (n=6) except for changes in low Beta vs changes in Thalamic BPND for the EO condition. Please note that the x-axes limits are different in the upper (subjects with TBI) vs lower (controls) panel.
